# Sodium-Glucose Transport Protein 2 (SGLT2) Inhibitors and the Risk of Pancreatitis: A Case Report

**DOI:** 10.7759/cureus.62957

**Published:** 2024-06-23

**Authors:** Zamanali Khakhar, Soraiya Manji, Ronak Kumar Patel, Sayed K Ali

**Affiliations:** 1 School of Medicine, University of Nairobi, Nairobi, KEN; 2 Department of Internal Medicine, Aga Khan University Hospital, Nairobi, KEN

**Keywords:** drug-induced acute pancreatitis, statins, diabetes mellitus type 2, acute pancreatitis, dapagliflozin, sglt2-inhibitors

## Abstract

Acute pancreatitis is a condition seldom encountered with the use of sodium-glucose cotransporter 2 (SGLT2) inhibitors. They are beneficial in the treatment of various conditions and offer great promise. Despite this, they are associated with several adverse effects, necessitating vigilance and further research. This case study reports a 69-year-old male with multiple comorbidities who presented with epigastric pain radiating to the back. Laboratory tests revealed elevated AST, ALT, GGT and lipase. The patient was diagnosed with acute pancreatitis secondary to the SGLT2 inhibitor therapy regimen. Cessation of dapagliflozin resulted in a complete resolution of symptoms. There is credible evidence to suggest the presence of an association between SGLT2 inhibitors and acute pancreatitis, although extensive research is warranted to consolidate this association.

## Introduction

Sodium-glucose cotransporter 2 (SGLT2) inhibitors are a novel category of medications employed in the treatment of type 2 diabetes mellitus (T2DM). With their recent development, SGLT2 inhibitors have already shown great promise in the management of T2DM and have become an integral part of the treatment strategy for this condition. Most of the glucose filtered by the glomeruli is reabsorbed at the proximal convoluted tubule via SGLT2. By targeting these transporters, SGLT2 inhibitors reduce the reabsorption of glucose in the kidneys hence promoting glycosuria and a resultant reduction in blood glucose levels. Research has also provided support for the use of SGLT2 inhibitors in T1DM, evidenced by reduced risk of hypoglycemia, weight loss, and lower insulin requirement with adjunctive therapy [1].

Dapagliflozin, empagliflozin and canagliflozin are some of the members of this drug class approved for the treatment of T2DM. In addition to their primary role in glucose regulation, SGLT2 inhibitors have demonstrated cardiovascular benefits which enables a more comprehensive approach to T2DM by using SGLT2 inhibitors. Studies have shown that SGLT2 inhibitors consistently lower blood pressure, with a more pronounced effect on systolic than diastolic pressure, without a compensatory increase in pulse rate [2]. They effectively reduce HbA1c levels, facilitate weight loss and retard the progression of cardiovascular disease and diabetic kidney disease by slowing the decline of renal function [3-4]. However, SGLT2 inhibitors may bring about various adverse effects such as diabetic ketoacidosis (DKA), urinary tract infections, acute kidney injury, and increased viscosity of blood by way of its diuretic action (haemoconcentration), which could in turn increase the risk of thromboembolism. Notably, the US Food and Drug Administration Adverse Event Reporting System (FAERS) has raised concerns regarding pancreatitis. However, this association has not yet been demonstrated in clinical trials. As their primary site of action within the proximal convoluted tubule, decreased filtration at the glomeruli, as occurs in chronic kidney disease, can significantly reduce the usefulness and efficacy of SGLT2 inhibitors [5].

Despite the significant benefits and overall usefulness of this drug class, it is crucial to evaluate the benefits and potentially rare adverse effects of SGLT2 inhibitors in certain high-risk groups.

Pancreatitis is commonly associated with cholelithiasis and alcohol (40-70% and 25-35%, respectively) [6]. Other causes of pancreatitis include hypertriglyceridemia, hypercalcemia, post-endoscopic retrograde cholangiopancreatography, viral infections (Coxsackie B, mumps) and trauma. Drug-induced pancreatitis, although infrequent, is most commonly attributable to the use of drugs such as mesalazine, azathioprine and statins and to a lesser degree SGLT2 inhibitors, though a WHO database lists 525 other different drugs that could possibly induce acute pancreatitis. Drug-induced pancreatitis accounts for 0.1% to 2% of all cases of pancreatitis [7-8]. It is commonly observed in those over the age of 40, but can also affect younger individuals, although it is rare. Combinations of various medications, excessive dosages or patient susceptibility may also contribute to the development of drug-induced pancreatitis. Data from case-control studies suggests that even drugs with concrete evidence for an association with pancreatitis only rarely cause the disease [8]. The symptoms usually resolve when the drug is discontinued. This would strongly indicate a causal association between pancreatitis and the drug in question.

We herein report a case of a 69-year-old male who developed pancreatitis following newly introduced SGLT2 inhibitor (dapagliflozin) therapy while other causes of pancreatitis were ruled out. The precise mechanisms by which SGLT2 inhibitors precipitate acute pancreatitis are poorly understood.

## Case presentation

A 69-year-old male with a history of T2DM for the last 20 years, bullous pemphigoid, dyslipidemia, chronic kidney disease and benign prostatic hyperplasia presented to our institution with complaints of recurrent episodes of bilious, non-bloody vomiting, coupled with epigastric pain radiating to the back for 72 hours. His symptoms had started at an airport terminal and he thought it was related to his intake of a sandwich while waiting for a connecting flight a few days prior to presentation. He denied any previous surgical procedures or previous episodes of pancreatitis. He sought regular care with his primary care physician whom he had recently seen about two weeks ago and due to his elevated HbA1c, had been started on dapagliflozin. He denied any recent surgeries or tobacco use. He consumed small amounts of alcohol socially once a week. He walked about 5 km daily as part of his exercise regimen. His medication regimen included a combination of metformin and sitagliptin twice a day, dapagliflozin 10 mg once a day (recently started), NPH (neutral protamine Hagedorn) insulin twice a day, cilnidipine 10 mg once a day, rosuvastatin 20 mg once a day, aspirin 81 mg once a day and a PPI. He had been compliant with all his medications, ensuring that he takes them daily as prescribed.

On presentation, his vital signs remained stable with a blood pressure of 130 over 86, a heart rate of 101, and a saturation of 93% on room air. On physical exam, he was non-icteric, with tenderness to palpation of his mid-epigastric region, without guarding or rebound tenderness. The rest of his examination was unremarkable. 

White blood cell count was 5.99 × 10^9^/L (normal: 4.5 to 11.0 × 10^9^/L), hemoglobin of 9.8 g/dl (normal: 14 to 18 g/dl), and platelets were shown to be 177 × 109/L (normal: 150 to 400 × 109/L).

His sodium was reported at 138 mmol/L (normal: 135 to 145 mmol/L), potassium at 3.99 mmol/l (normal: 3.6 to 5.2 mmol/L), and creatinine at 156 µmol/L (normal: 61.9 to 114.9 µmol/L) with a creatinine clearance which measures 38 mL/min (normal: 110 to 150 mL/min).

His labs showed a raised AST at 66 U/L (normal: 8 to 48 U/L), ALT 101 U/L (normal: 7 to 55 U/L), alkaline phosphatase at 166 U/L (normal: 40 to 129 U/L) with a GGT of 131 U/L (normal: 8 to 61 U/L). His lipase was also elevated at 1566 U/L (normal: 0 to 160 U/L). His electrolytes remained within normal limits. A lipid panel showed a triglyceride level of 2.1 mmol/L (normal: less than 1.7 mmol/L). HbA1c was reported at 7.8% indicating diabetes mellitus (normal is less than 6.5%) and his calcium levels were normal.

**Figure 1:**
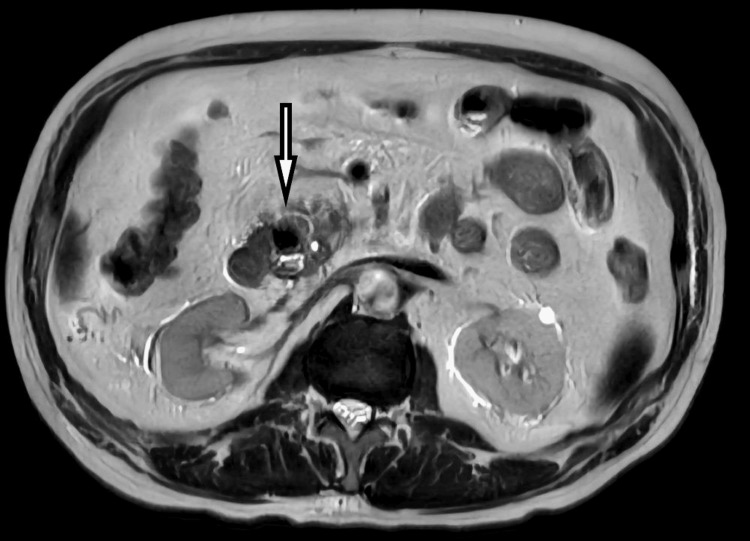
Magnetic resonance cholangiopancreatography shows restricted diffusion at the head and neck of the pancreas suggestive of acute pancreatitis without any necrosis or collection (white arrow).

An urgent right upper ultrasound revealed sonographic features suggestive of pancreatitis and cholecystitis without sonographic evidence of cholelithiasis. A CT scan of his abdomen showed similar findings as well without any collection of pseudocysts. Magnetic resonance cholangiopancreatography showed a restricted diffusion at the head and neck of the pancreas suggestive of acute pancreatitis without any necrosis or collection (Figure 1).

## Discussion

The current case describes a patient who developed acute pancreatitis one week after initiating treatment with dapagliflozin for the management of T2DM. The significance of this report is to highlight the potentially rare adverse effects of a widely prescribed drug, underscoring crucial considerations for the use of this drug in common clinical practice. The precise mechanisms by which SGLT2 inhibitors precipitate acute pancreatitis are poorly understood. It is postulated that SGLT2 inhibitors decrease the kidney’s capacity to reabsorb glucose, resulting in a lower plasma glucose level. This disruption in glucose metabolism can lead to inflammation and injury of pancreatic tissue. The suggested mechanisms for how SGLT2 inhibitors may cause pancreatitis include direct toxicity to pancreatic cells, immune-mediated responses, or idiosyncratic reactions [9].

Acute pancreatitis associated with the use of SGLT2 inhibitors is a rare adverse effect of this drug class but it has been documented in the past. Gutch et al. described a case of a 48-year-old man with T2DM who was resistant to metformin upon which dapagliflozin was added to the regimen. Within three days, there was onset of diffuse abdominal pain, nausea, and vomiting. By the fourth day, he was diagnosed with acute pancreatitis and DKA. After ruling out the common causes, the temporal relationship suggested acute pancreatitis precipitated by dapagliflozin. Discontinuation resulted in the resolution of symptoms [10]. Sujanani et al. also reported a similar case where the addition of dapagliflozin to the daily regimen of a patient with T2DM resulted in the development of acute pancreatitis [11]. Most cases of drug-induced pancreatitis present with mild to moderate severity, however, some cases may lead to fatal complications [12].

The present case describes the development of acute pancreatitis in a 69-year-old male of Indian descent with multiple comorbidities within one week of initiation of dapagliflozin. A diagnosis of acute pancreatitis was established after excluding other probable etiologies of acute pancreatitis including normal triglycerides and calcium levels. Following discontinuation, the patient recovered with complete resolution of symptoms therefore substantiating the association between dapagliflozin therapy and acute pancreatitis. The patient's use of rosuvastatin, along with other medications, raises concerns about the potential role of these drugs in the development of pancreatitis. Logistic regression analysis by Zhang et al. identified that different SGLT2 inhibitors and their combinations with statins were independent risk factors for acute pancreatitis mortality in the patients [13]. However, the patient had been taking these medications for a considerable period without experiencing complaints or symptoms of acute pancreatitis, thus suggesting that these agents were unlikely to be the cause of the current diagnosis. Nevertheless, it is important to consider the potential of an additive or synergistic interaction between these agents and dapagliflozin, which could contribute to the onset of pancreatitis [14]. The race of the patient may have some contribution to the development of drug-induced pancreatitis. However, this relationship is not yet well-established and requires necessary additional research to confirm whether race significantly impacts the susceptibility to drug-induced pancreatitis.

Increased awareness of new drug classes and their associated pharmacodynamics is necessary for the immediate identification and management of rare adverse effects in order to prevent their progression and facilitate swift resolution. Therefore, monitoring patients for unusual adverse effects, in this case, signs of pancreatitis is crucial. This can be achieved with regular check-ups and education on recognizing the adverse effects that may be expected while using the drug.

A drug rechallenge test involves the administration of a previously withdrawn drug to assess the causal relationship between the medication and the outcome. We acknowledge that a drug rechallenge test was not performed, which would further confirm the suspicion of acute pancreatitis due to dapagliflozin. Notably, it remains to be determined whether the observed pancreatitis is solely attributable to SGLT2 inhibitors or from the combined therapy regimen in this case and whether a genetic predisposition may contribute to the development of adverse drug reactions in certain individuals more than others.

Our patient did well post cessation of his SGLT2 inhibitor (dapagliflozin) and was discharged 72 hours later once he was able to tolerate a regular diet. We advised him to stop the use of SGLT2 inhibitors. On follow-up two weeks later, the patient was doing relatively well, with no symptoms, and had commenced his exercise regimen.

## Conclusions

While SGLT2 inhibitors offer significant benefits and show great promise in their multifaceted approach to the management of T2DM and heart failure by virtue of their cardiovascular benefits, they have the potential to cause rare adverse effects, most notably, in some cases, pancreatitis. While the exact mechanisms of drug-induced pancreatitis due to SGLT2 inhibitors remain unclear, increased vigilance is required with the use of such drugs through constant monitoring of patients and regular check-ups. This case highlights the need for further research to explore the underlying mechanisms and pathophysiology of the rare adverse outcomes associated with SGLT2 inhibitors and to identify risk factors, including racial and genetic predispositions. Additional research should aim to provide more robust evidence on the relationship between SGLT2 inhibitors and pancreatitis in order to improve patient outcomes.
